# Evolution and Effects of Ad Hoc Multidisciplinary Team Meetings in the Emergency Intensive Care Unit: A Five-Year Analysis

**DOI:** 10.3390/jcm13154324

**Published:** 2024-07-24

**Authors:** Tetsuya Yumoto, Takashi Hongo, Takafumi Obara, Kohei Ageta, Toshiyuki Aokage, Kohei Tsukahara, Atsunori Nakao, Hiromichi Naito

**Affiliations:** Department of Emergency, Critical Care, and Disaster Medicine, Faculty of Medicine, Dentistry, and Pharmaceutical Sciences, Okayama University, Okayama 700-8558, Japan; taka.hongo123@gmail.com (T.H.); dainosinn@gmail.com (T.O.); ageage1982@gmail.com (K.A.); s8001@nms.ac.jp (T.A.); hei.trp@gmail.com (K.T.); qq-nakao@okayama-u.ac.jp (A.N.); naito05084@gmail.com (H.N.)

**Keywords:** clinical conference, end-of-life care, ICU rounds, multidisciplinary, team meetings

## Abstract

**Background**: Multidisciplinary team meetings (MDTMs) are crucial in the ICU. However, daily rounds may not address all sensitive issues due to time constraints and the complexity of cases. This study aimed to describe detailed information and characteristics of ad hoc MDTMs in the ICU. **Methods**: This single-center, retrospective study analyzed adult emergency ICU admissions at Okayama University Hospital from 1 January 2019 to 31 December 2023. During this period, weekly regular multidisciplinary team ICU rounds were introduced in June 2020, and regular weekday morning MDTMs began in April 2022. A multiple logistic regression analysis was applied to determine the impact of these changes on the frequency of ad hoc MDTMs, adjusting for variables including annual changes. **Results**: The study analyzed 2487 adult EICU patients, with a median age of 66, and 63.3% of them male. MDTMs were held for 168 patients (6.8%), typically those with severe conditions, including higher COVID-19 prevalence and APACHE II scores, and longer ICU stays. Despite a constant total number of MDTMs, the likelihood of conducting ad hoc MDTMs increased annually (adjusted OR 1.19; 95% CI, 1.04–1.35). Of the 329 MDTMs conducted for these patients, 59.0% addressed end-of-life care, involving an average of 11 participants, mainly nurses and emergency and critical-care physicians. **Conclusions**: Changes in ICU round and meeting structures might be associated with a higher frequency of conducting ad hoc MDTMs, highlighting their evolving role and importance in patient care management.

## 1. Introduction

A multidisciplinary team approach is currently recognized as a fundamental and essential aspect in the ICU, ensuring comprehensive patient care through the integration of diverse expertise and experience from various healthcare professionals [1]. Multidisciplinary team meetings (MDTMs), which include daily rounds, ad hoc meetings, and occasional virtual sessions, have been extensively documented to enhance patient outcomes, improve cost-efficiency, and foster better communication among healthcare providers and with families [2,3,4,5,6,7].

Daily ICU rounds allow critical care professionals to set the patient’s individualized goal of care and facilitate team collaboration and coordination. However, the conventional structure of daily ICU rounds, while effective for routine patient management, sometimes falls short of addressing more complex, sensitive issues. For instance, discussions surrounding end-of-life care decisions or the resolution of interprofessional conflicts may require a more focused, in-depth dialogue than what daily rounds typically allow [4]. Constraints such as time limitations, the unpredictable nature of clinical emergencies, and the inherent complexity of patients’ medical conditions can hinder the ability to engage in these critical conversations thoroughly. In certain situations, therefore, ad hoc MDTMs serve as a pivotal role in facilitating shared decision making and achieving goals [8].

Ad hoc MDTMs are convened as needed, to address specific patient issues that arise unexpectedly, requiring immediate multidisciplinary input. These meetings are particularly valuable for making rapid decisions in complex cases and ensuring that all aspects of patient care are considered [8]. The advent of telemedicine has facilitated virtual MDTMs, allowing for broader participation and the inclusion of specialists who may not be physically present. These meetings have proven effective in maintaining continuity of care and ensuring timely decision-making [9]. Beyond daily rounds, scheduled weekly or monthly meetings allow for in-depth review and planning for patients with prolonged ICU stays or those with particularly challenging conditions. These sessions provide an opportunity to reassess care plans, involve family members, and ensure that all team members are aligned in their approach [10]. Research has consistently shown the benefits of MDTMs in the ICU. Studies have demonstrated that these meetings can reduce mortality rates, enhance the quality of care, and improve the health-related quality of life for patients post-discharge [2,11,12]. Additionally, structured communication strategies, particularly those focused on end-of-life care, have been linked to reduced burnout among ICU staff, highlighting the holistic benefits of multidisciplinary collaboration [13,14].

Despite the growing recognition of their importance, there is a notable gap in the literature regarding the specifics of ad hoc MDTMs within the ICU context, especially concerning their operational dynamics, frequency and objectives, and the composition of participants. It would be of considerable interest to elucidate and document the emerging significance of these meetings. This study aimed to shed light on these aspects by offering a comprehensive analysis of ad hoc emergency multidisciplinary team meetings in the ICU, thereby contributing to a deeper understanding of their value and implications for critical care practices.

## 2. Materials and Methods

### 2.1. Ethics

The present study received approval from the Okayama University Hospital Ethics Committee (reference number K2403-005) and was conducted in accordance with the principles outlined in the Declaration of Helsinki. Given the retrospective nature of the study and using anonymous data, the requirement for written informed consent was waived by the Ethics Committee.

### 2.2. Study Design and Setting

This retrospective, single-center study was conducted in the Emergency ICU (EICU) at Okayama University Hospital. The EICU is a closed unit with 12 beds dedicated exclusively to emergency patients from outside the hospital, including 2 beds designated for COVID-19 patients, and is managed by emergency and critical-care physicians. The study included adult patients aged 18 years or older who were admitted to the EICU between 1 January 2019 and 31 December 2023.

### 2.3. Changes in EICU Round and Meetings

**Figure 1:**
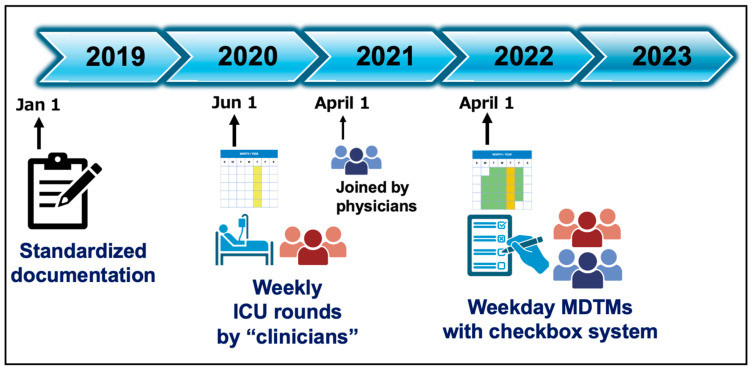
Timeline of EICU rounds and meeting structure. The illustration presenting the evolution of EICU rounds and meetings, starting with the adoption of standardized documentation on 1 January 2019. Key developments include the start of weekly clinician-led rounds on 1 June 2020, physician participation from 1 April 2021, and the introduction of checkbox-based morning MDTMs on 1 April 2022. EICU: emergency intensive care unit, MDTMs: multidisciplinary team meetings.

Changes in the EICU round and meeting structure are illustrated in Figure 1, which began with the initiation of standardized documentation by a nurse regarding multidisciplinary rounds or meetings within the electronic medical record on 1 January 2019. This documentation framework included listing the members present, outlining the objectives of the meetings, and providing context based on Jonsen’s four-box model for medical decision-making, when necessary [15]. From 1 June 2020, weekly ICU rounds by clinicians, excluding physicians, were implemented. These rounds initially involved healthcare professionals other than physicians, to allow for a focused assessment on the nursing and supportive-care aspects of patient management. This approach aimed to strengthen the collaboration among these team members, ensuring that all perspectives were integrated into patient care plans. Subsequently, it was determined that the inclusion of physicians would further enhance the multidisciplinary approach with their medical expertise. Therefore, physicians joined these rounds on 1 April 2021. This phased inclusion allowed for a smoother integration of physicians into the established structure, fostering a more cohesive and collaborative environment. In addition, morning MDTMs, utilizing a checkbox system, were initiated on 1 April 2022. These half-hour sessions were designed to briefly discuss the daily goals of care for each patient in the ICU. The multidisciplinary team comprised emergency and critical-care physicians, nurses, pharmacist, a clinical engineer, a nutritionist, a physical therapist, and a medical social worker.

### 2.4. Data Collection

The medical records of individuals were thoroughly reviewed to retrieve information on age, sex, primary disease or injury, Acute Physiology and Chronic Health Evaluation (APACHE) II score, the holding of ad hoc MDTMs, determination of end-of-life status, brain death during the ICU stay, organ donation, length of ICU stay, and outcomes at ICU discharge. If the record indicated that an ad hoc MDTM was held, the data collected included the number of meetings for each patient and the attendance of clinician members at each meeting. We also collected the primary themes of MDTMs in the ICU, which were categorized into discussions regarding end-of-life considerations, sharing patient information, and collaborative care planning.

### 2.5. Outcomes

The primary outcome was the occurrence of ad hoc MDTMs. Secondary outcomes included the in-hospital mortality, length of hospital stay, themes of the MDTMs, the number of participants, and the composition of clinician members within the ad hoc MDTMs.

### 2.6. Statistical Analyses

Continuous data were presented as medians with interquartile range (IQR), and categorical data as counts and percentages. The Chi-square test was utilized for categorical variables. For continuous data, the Mann–Whitney U test was applied to compare two groups, while the Kruskal–Wallis test was used for multiple group comparisons. A multiple logistic regression analysis was used to calculate the adjusted odds ratios (ORs) and 95% confidence intervals (CIs) for the likelihood of conducting ad hoc MDTMs. This analysis included variables such as age, sex, APACHE II score, COVID-19 status, and ICU length of stay, reflecting the hypothesis that patients with higher APACHE II scores or longer ICU stays, including those with COVID-19, are more likely to necessitate ad hoc MDTMs [12]. Additionally, the year (the per-1-year increase) was included in the variable to assess changes in the structure of rounds and meetings in the EICU from 2019 to 2023. To examine the effect of the number of MDTMs on in-hospital mortality, a multiple logistic regression analysis was performed, adjusting for age, sex, APACHE II scores, end-of-life status, admission year, and the number of MDTMs. Similarly, to examine the effect of the number of MDTMs on hospital length of stay, a multiple linear regression analysis was conducted, adjusting for the same variables, with results presented as β coefficient with 95% CI. For sensitivity analysis, the study period was divided into three phases: Phase I, from 1 January 2019 to 31 May 2020; Phase II, from 1 June 2020 to 31 March 2022; and Phase III, from 1 April 2022 to 31 December 2023 (Figure 1). ‘Year’ was then substituted by ‘Phase’, using Phase I as the reference category. All tests were two-tailed, and a *p* value of <0.05 was considered statistically significant. The analyses were conducted using IBM SPSS Statistics 26 (IBM SPSS, Chicago, IL, USA).

## 3. Results

During the 5-year study period, a total of 2757 patients were admitted to the EICU. After excluding patients aged 18 or younger, 2487 adult patients were included in the analysis.

Table 1 shows the baseline characteristics of the study population based on the year of EICU admission. The overall median age was 66 years (IQR, 47–77); 1575 (63.3%) were male. Trauma was the most common primary disease or injury, followed by pneumonia/sepsis/infectious diseases, stroke, post-cardiac arrest syndrome, and intoxication. The median APACHE II score across the cohort was 19 (IQR, 12–26). Over the course of the study, the median length of stay in the EICU tended to decrease. Overall, ICU morality was 6.6% (163/2487). MDTMs were conducted for 168/2487 patients (6.8%). There was no statistically significant difference in the percentage of ad hoc MTDMs among the years studied.

Table 2 compares the baseline characteristics of patients with and without MDTMs. Those who underwent MDTMs had a lower prevalence of trauma and a higher prevalence of post-cardiac arrest syndrome, COVID-19, higher APACHE II scores, longer ICU stays, and higher ICU mortality.

Table 3 presents the crude and adjusted odds ratios (ORs) for the occurrence of ad hoc MDTMs. After adjusting for covariates, each additional year was associated with an increased likelihood of conducting ad hoc MDTMs (adjusted OR, 1.19; 95% CI, 1.04–1.35).

A multiple logistic regression analysis revealed that the number of MDTMs was not associated with in-hospital mortality (adjusted OR, 0.83; 95% CI, 0.64–1.07). However, a multiple linear regression analysis showed that the number of MDTMs was associated with the length of hospital-stay days (β coefficient, 5.54; 95% CI, 4.36–6.73).

For 168 patients, a total of 329 MDTMs were conducted. Table 4 presents trends in the themes and the composition of participants in these MDTMs from 2019 to 2023. A significant focus of these meetings, with 194 (59.0%) overall, was on end-of-life care, with the highest prevalence observed in 2019. The results suggest a notable shift in the objectives of MDTMs from end-of-life care considerations to more collaborative care planning and patient information sharing over the years. The median number of participants across these meetings was 11 (IQR, 8–14). Emergency and critical-care physicians and nurses formed the core of these meetings, with their numbers slightly fluctuating but fundamentally showing a strong and consistent presence throughout the period. Specifically, emergency and critical-care physicians were present in a median number of 2 (IQR, 2–3), and nurses in a median number of 8 (IQR, 6–10), emphasizing their pivotal role in MDTMs. Participation by other clinicians, encompassing a diverse group ranging from psychiatrists to clinical engineers, was less frequent but nonetheless integral, accounting for an essential component of the meetings. For instance, clinical engineers were those with the highest participation rate among other clinicians, at 12.8%. The involvement of other specialized roles such as physical therapists, pharmacists, and medical social workers, varied over the years, reflecting the adaptive and situational requirements of the ICU environment.

As for sensitivity analysis, Phase II and Phase III were associated with an increased likelihood of conducting ad hoc MDTMs (Phase II: adjusted OR, 1.84; 95% CI, 1.11–3.04, Phase III: adjusted OR, 2.03; 95% CI, 1.24–3.33), considering Phase I as a reference.

## 4. Discussion

In this 5-year study, we observed that the actual number of MDTMs remained constant. However, after adjusting for covariates related to severity, factors likely to require ad hoc MDTMs, alterations in the regular structure of ICU rounds and meetings were associated with an increased likelihood of conducting ad hoc MDTMs. The vast majority of these ad hoc MDTMs, primarily focused on end-of-life care, were led by nurses, along with emergency and critical-care physicians.

A previous analysis highlighted the fact that daily multidisciplinary ICU rounds could significantly reduce mortality among medical ICU patients [2]. However, mortality alone may not fully capture the quality of care in the ICU. In certain situations, focusing on enhancing the health-related quality of life post-ICU discharge, optimizing end-of-life care, alleviating family or caregivers’ burdens, and even improving ICU staff well-being, becomes paramount [11]. Given the high mortality and morbidity inherent in the ICU setting, healthcare professionals often encounter ethically and emotionally demanding scenarios [13]. Notably, the adoption of intensive communication strategies centered on a multidisciplinary approach regarding end-of-life practices has been linked to the mitigation of burnout syndrome, underscoring the multifaceted benefits of such collaborations [14].

Our findings suggest that while the frequency of MDTMs did not directly impact mortality, they were associated with longer hospital stays. This likely reflects the need for more extensive discussions in complex or severe cases. Our results indicate that more MDTMs do not inherently reduce mortality but may improve other aspects of patient care, such as discharge planning and coordination. Further research is needed to determine if MDTMs enhance the quality-of-care experiences for patients and families and the staff well-being.

Reflecting on global trends in ICU care, the escalating frequency of ad hoc MDTMs aligns with the growing appreciation for the multidisciplinary approach’s advantages, especially in managing severe cases and navigating end-of-life situations [16]. The focused attention on individual patient needs, particularly regarding end-of-life care, highlights the need for more frequent and specific interdisciplinary collaborations, an area where ad hoc MDTMs are particularly effective.

**Figure 2:**
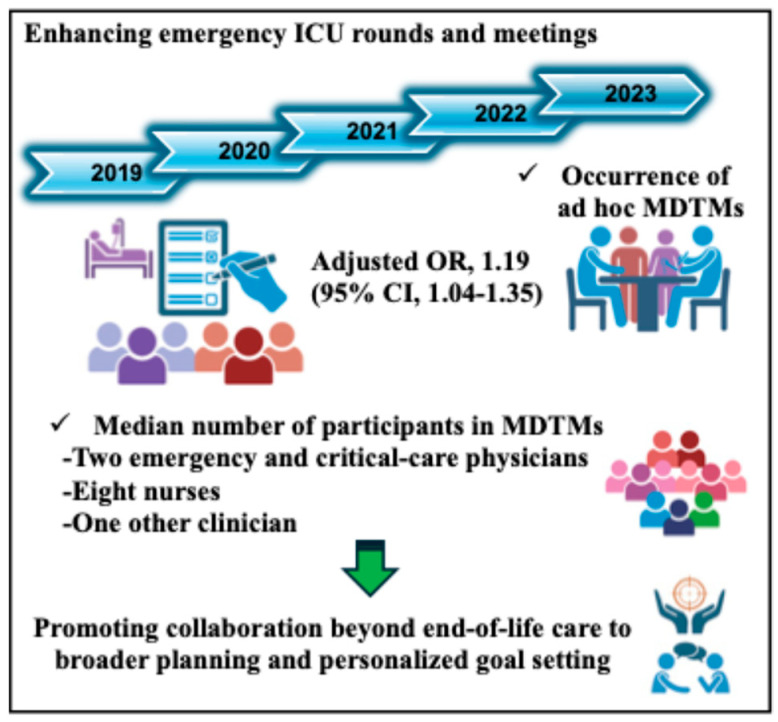
Effects of implementing structural modifications to regular emergency ICU rounds and meetings. An OR of 1.19 indicates that with each additional year, the odds of conducting ad hoc MDTMs increased by 19%. ICU: intensive care unit, OR: odds ratio, CI: confidence interval, MDTMs: multidisciplinary team meetings.

Moreover, the intensified likelihood of MDTMs in the latter phases of our study suggests a refinement over time in recognizing scenarios that benefit from ad hoc discussions. This evolution likely stems from integrating changes into regular ICU meetings, thereby facilitating more comprehensive interdisciplinary exchanges, creating environments for discussing complex cases, and enabling deeper exploration of patient-care goals beyond the daily rounds’ limitations (Figure 2).

We recognize the importance of inclusive interdisciplinary communication. Initially, physicians were excluded from ad hoc meetings to focus on nursing and supportive care. Moving forward, we plan to include specific sub ad hoc groups with physician agreement and re-evaluate the structure to include more frequent team meetings and better planning, facilitating effective multidisciplinary discussions.

While a former survey from Germany detailed the structure and members of rounding teams in the ICU [17], it is crucial to differentiate the fact that our focus on ad hoc MDTMs diverges from routine ICU rounds. This distinction is vital, as our findings highlight the unique contributions of ad hoc MDTMs to the complex landscape of ICU care. This detailed analysis of the MDTMs which was conducted highlights the dynamic and complex nature of multidisciplinary care in the ICU, emphasizing the critical role of a wide range of healthcare professionals in addressing particularly challenging aspects of patient care, such as end-of-life decisions.

This study has several limitations. First, it did not collect information on comorbidities, frailty, or social backgrounds, including family dynamics, which could influence or trigger the need for MDTMs and which represent crucial factors in patient care [18]. Second, since the data were extracted from medical records, patients without documented MDTMs were not explored, although this approach reflects real-world practices. Third, the outcomes for patients, families, or clinicians post-MDTM were not collected, limiting insights into the effectiveness of these meetings. Fourth, the specific content of discussions, including any conflicts among clinicians, was not examined in detail [19]. Lastly, the single-center, retrospective design limits the generalizability of our findings to other institutions, regions, or countries. Despite these limitations, this study is the first to report on the occurrence and nature of ad hoc MDTMs in the ICU, offering valuable insights into their role and implementation.

## 5. Conclusions

Our study indicates that structural modifications to ICU rounds and meetings could potentiate the multidisciplinary collaboration crucial for high-stakes decision-making. Over the years, there has been a shift from end-of-life care considerations to more collaborative care planning and patient information sharing. These adjustments were associated with an increased likelihood of conducting ad hoc MDTMs, underscoring their evolving role in patient-care management. This trend highlights the need for systemic changes to facilitate effective multidisciplinary discussions, enhancing overall patient-care quality and coordination. Regular ICU rounds can help identify cases requiring further discussion in ad hoc MDTMs, extending their benefits beyond end-of-life care to broader collaborative care planning and information sharing. Future research should delve into the direct impact of these structural changes on the outcomes of patients, families, and clinicians, further affirming the need for adjustments within ICU care protocols.

## Figures and Tables

**Table 1:** Baseline characteristics of the study population based on the year of emergency ICU admission.

	Overall n = 2487	2019 n = 383	2020 n = 406	2021 n = 431	2022 n = 663	2023 n = 604	*p* Value
Age, median (IQR), yr	66 (47, 77)	62 (41, 75)	63 (46, 75)	64 (44, 77)	70 (51, 79)	66 (49, 79)	<0.001
Male sex, n (%)	1575 (63.3)	240 (62.7)	245 (60.3)	270 (62.6)	434 (65.5)	386 (63.9)	0.541
Primary disease or injury, n (%)							<0.001
Trauma	980 (39.4)	166 (43.3)	187 (46.1)	182 (42.2)	224 (33.8)	221 (36.6)	
Pneumonia/sepsis/infectious diseases	340 (13.7)	39 (10.2)	55 (13.5)	63 (14.6)	94 (14.2)	89 (14.7)	
Stroke	235 (9.4)	37 (9.7)	18 (4.4)	36 (8.4)	81 (12.2)	63 (10.4)	
Post-cardiac arrest syndrome	156 (6.3)	30 (7.8)	34 (8.4)	28 (6.5)	43 (6.5)	21 (3.5)	
Intoxication	135 (5.4)	24 (6.3)	17 (4.2)	18 (4.2)	41 (6.2)	35 (5.8)	
Neurological	116 (4.7)	17 (4.4)	15 (3.7)	23 (5.3)	28 (4.2)	33 (5.5)	
Metabolism	110 (4.4)	18 (4.7)	8 (2.0)	12 (2.8)	37 (5.6)	35 (5.8)	
Heat stroke or hypothermia	75 (3.0)	6 (1.6)	8 (2.0)	10 (2.3)	38 (5.7)	13 (2.2)	
Respiratory	63 (2.5)	10 (2.6)	13 (3.2)	8 (1.9)	14 (2.1)	18 (3.0)	
Cardiac	59 (2.4)	7 (1.8)	8 (2.0)	14 (3.2)	13 (2.0)	17 (2.8)	
Burn injury	45 (1.8)	5 (1.3)	11 (2.7)	11 (2.6)	9 (1.4)	9 (1.5)	
Gastrointestinal bleeding	44 (1.8)	3 (0.8)	3 (0.7)	5 (1.2)	14 (2.1)	19 (3.1)	
Others	128 (5.1)	20 (5.2)	29 (7.1)	21 (4.9)	27 (4.1)	31 (5.1)	
COVID-19, n (%)	84 (3.4)	0 (0)	19 (4.7)	19 (4.4)	21 (3.2)	25 (4.1)	0.001
APACHE II, median (IQR)	19 (12, 26)	18 (12, 29)	18 (12, 28)	18 (12, 25)	19 (12, 26)	18 (13, 25)	0.336
End-of-life stage, n (%)	118 (4.7)	21 (5.5)	26 (6.4)	13 (3.0)	28 (4.2)	24 (4.0)	0.026
Brain death, n (%)	54 (2.2)	9 (2.3)	11 (2.7)	5 (1.2)	17 (2.6)	12 (2.0)	0.515
Organ donation after brain death, n (%)	18 (0.7)	2 (0.5)	3 (0.7)	3 (0.7)	3 (0.5)	7 (1.2)	0.646
ICU length of stay, median (IQR), days	4 (2, 9)	5 (2, 13)	4 (2, 11)	3 (2, 9)	3 (2, 7)	3 (2, 6)	<0.001
Outcome at ICU discharge, n (%)							<0.001
Death	163 (6.6)	31 (8.1)	28 (6.9)	24 (5.6)	47 (7.1)	33 (5.5)	
Transfer to another hospital	1357 (54.6)	184 (48.0)	189 (46.6)	226 (52.4)	391 (59.0)	367 (60.8)	
Move to a ward	557 (22.4)	88 (23.0)	120 (29.6)	103 (23.9)	134 (20.2)	112 (18.5)	
Discharge to home	406 (16.3)	78 (20.4)	68 (16.7)	78 (18.1)	91 (13.7)	91 (15.1)	
Ad hoc MDTMs, n (%)	168 (6.8)	25 (6.5)	36 (8.9)	22 (5.1)	47 (7.1)	38 (6.3)	0.278

IQR: interquartile range, APACHE: Acute Physiology and Chronic Health Evaluation, MDTMs: multidisciplinary team meetings.

**Table 2:** Comparison of baseline characteristics between patients with and without ad hoc MDTMs.

	**(+) MDTMs** **n = 168**	**(−) MDTMs** **n = 2319**	***p*** **Value**
Age, median (IQR), yr	65 (49, 76)	67 (47, 77)	0.769
Male sex, n (%)	109 (64.9)	1466 (63.2)	0.666
Primary disease or injury, n (%)			<0.001
Trauma	24 (14.3)	956 (41.2)	
Pneumonia/sepsis/infectious diseases	31 (18.5)	309 (13.3)	
Stroke	20 (11.9)	215 (9.3)	
Post-cardiac arrest syndrome	64 (38.1)	92 (4.0)	
Intoxication	0 (0)	135 (5.8)	
Neurological	4 (2.4)	112 (4.8)	
Metabolism	3 (1.8)	107 (4.6)	
Heat stroke or hypothermia	7 (4.2)	68 (2.9)	
Respiratory	6 (3.6)	57 (2.5)	
Cardiac	2 (1.2)	57 (2.5)	
Burn injury	4 (2.4)	41 (1.8)	
Gastrointestinal bleeding	0 (0)	44 (1.9)	
Others	3 (1.8)	125 (5.4)	
COVID-19, n (%)	15 (8.9)	69 (3.0)	0.001
APACHE II, median (IQR)	31 (26, 37)	18 (12, 24)	<0.001
End-of-life stage, n (%)	103 (61.3)	9 (0.4)	<0.001
Brain death, n (%)	51 (30.4)	3 (0.1)	<0.001
Organ donation after brain death, n (%)	18 (10.7)	0 (0)	<0.001
ICU length of stay, median (IQR), days	11 (5, 17)	3 (1, 7)	<0.001
Outcome at ICU discharge, n (%)			<0.001
Death	90 (53.6)	73 (3.1)	
Transfer to another hospital	65 (38.7)	1292 (55.7)	
Move to a ward	11 (6.5)	546 (23.5)	
Discharge to home	2 (1.2)	408 (17.6)	

MDTMs: multidisciplinary team meetings, IQR: interquartile range, APACHE: Acute Physiology and Chronic Health Evaluation.

**Table 3:** Multiple logistic regression analyses to estimate adjusted effects of yearly trend on the number of MDTMs.

Variables	Crude OR	95% CI	Adjusted ORs	95% CI
Age, yr	1.00	0.99–1.01	0.98	0.97–0.99
Male	1.08	0.77–1.49	1.14	0.79–1.67
COVID-19	3.20	1.79–5.72	4.29	2.13–8.65
APACHE II	1.16	1.14–1.19	1.18	1.15–1.21
ICU length of stay, days	1.06	1.04–1.07	1.03	1.01–1.04
Year (per 1-year increase)	0.97	0.87–1.08	1.19	1.04–1.35

MDTMs: multidisciplinary team meetings, ORs: odds ratios, APACHE: Acute Physiology and Chronic Health Evaluation.

**Table 4:** Trends in objectives and participant composition of ad hoc MDTMs from 2019 to 2023.

	Overall n = 329	2019 n = 51	2020 n = 80	2021 n = 69	2022 n = 71	2023 n = 58	*p* Value
Themes, n (%)							<0.001
End-of-life care considerations	194 (59.0)	48 (94.1)	50 (62.5)	30 (42.5)	40 (56.3)	26 (44.8)	
Collaborative care planning	71 (21.6)	0 (0)	4 (5.0)	16 (23.2)	25 (35.2)	26 (27.6)	
Only sharing patient information	64 (19.4)	3 (5.9)	26 (32.5)	23 (33.3)	6 (8.5)	6 (10.3)	
A total number of participants per meeting, median (IQR)	11 (8, 14)	13 (10, 16)	11 (8, 14)	13 (10, 16)	9 (7, 11)	9 (6, 12)	<0.001
Emergency and critical care physicians	2 (2, 3)	3 (2, 3)	2 (2, 3)	2 (1, 3)	2 (2, 2)	2 (1, 3)	<0.001
Nurses	8 (6, 10)	9 (6, 12)	8 (6, 11)	9 (7, 12)	7 (4, 9)	6 (4, 9)	<0.001
Other clinicians	0 (0, 1)	1 (0, 2)	0 (0, 1)	0 (0, 2)	0 (0, 0)	0 (0, 1)	0.003
Participation rate of other clinicians, n (%)							
Psychiatrist	21 (6.4)	2 (3.9)	0 (0)	1 (1.4)	4 (5.6)	2 (3.4)	0.262
Physicians of other subjects	21 (6.4)	6 (11.8)	2 (2.5)	7 (10.1)	3 (4.2)	3 (5.2)	0.141
Resident	27 (8.2)	7 (13.7)	9 (11.3)	5 (7.2)	3 (4.2)	3 (5.2)	0.259
Medical student	9 (2.7)	4 (7.8)	0 (0)	3 (4.3)	0 (0)	2 (3.4)	0.041
Clinical engineer	42 (12.8)	15 (29.4)	13 (16.3)	9 (13.0)	2 (2.8)	3 (5.2)	<0.001
Physical therapist	26 (7.9)	2 (3.9)	8 (10.0)	13 (18.8)	1 (1.4)	2 (3.4)	0.001
Pharmacist	24 (7.3)	3 (5.9)	9 (11.3)	9 (13.0)	1 (1.4)	2 (3.4)	0.036
Medical social worker	12 (3.6)	1 (2.0)	0 (0)	1 (1.4)	4 (5.6)	6 (10.3)	0.014
Ethical consultation team	10 (3.0)	0 (0)	1 (1.3)	1 (1.4)	4 (5.6)	4 (6.9)	0.101
Nutritionist	9 (2.7)	0 (0)	3 (3.8)	5 (7.2)	1 (1.4)	0 (0)	0.059
Organ transplant coordinator	9 (2.7)	2 (3.9)	2 (2.5)	0 (0)	1 (1.4)	4 (6.9)	0.162
Psychologist	4 (1.2)	0 (0)	0 (0)	2 (2.9)	0 (0)	2 (3.4)	0.165
Others ^a^	6 (1.8)	0 (0)	0 (0)	3 (4.3)	0 (0)	3 (5.2)	<0.001

^a^ Other clinicians refers to healthcare professionals who are neither emergency and critical-care physicians nor nurses. MDTMs: multidisciplinary team meetings, IQR: interquartile range.

## Data Availability

The datasets from this study are available from the corresponding author upon reasonable request.
